# The effect of physical activity on fertility: a mini-review

**DOI:** 10.1016/j.xfre.2023.04.005

**Published:** 2023-04-14

**Authors:** Minhal Mussawar, Ashley A. Balsom, Julia O. Totosy de Zepetnek, Jennifer L. Gordon

**Affiliations:** a Department of Psychology, University of Regina, Regina, Saskatchewan, Canada; b Kinesiology and Health Studies, University of Regina, Regina, Saskatchewan, Canada

**Keywords:** Infertility, physical activity, exercise, ovulation, assisted reproductive technologies

## Abstract

Although lifestyle factors such as diet, cigarette smoking, and alcohol consumption are increasingly recognized as important contributors to the risk of subfertility, the role of exercise in fertility remains less clear. As such, it is challenging for healthcare providers to deliver clear, evidence-based recommendations to patients regarding the optimal frequency and intensity with which they should exercise to maximize their chances of conception. Therefore, this review provides a critical overview of the available research for various patient populations.

## Highlights

• Overweight and obese women with polycystic ovary syndrome should be encouraged to engage in vigorous aerobic exercise or resistance training to optimize their chances of conceiving.
• In women undergoing assisted reproduction for other diagnoses, exercise likely has little to no impact on treatment outcomes. Individuals can therefore continue their regular exercise regimen throughout the treatment.
• In healthy women who are trying to conceive, the potential for regular vigorous exercise to negatively impact fertility should be considered. The presence of anovulation and/or a luteal phase defect caused by vigorous exercise should be evaluated.

The American Society for Reproductive Medicine provides clear guidance regarding the impact of smoking, alcohol consumption, and diet on conception, coupled with appropriate patient recommendations (1). However, no such guidelines exist regarding patient engagement in physical activity (PA) and exercise, making it challenging for healthcare providers to deliver clear, evidence-based recommendations regarding the optimal frequency and intensity with which patients should exercise to maximize their chances of conception. This is particularly true given that appropriate recommendations may differ according to a patient’s baseline fitness level, health status, and infertility-related diagnosis. Thus, this review aims to provide an up-to-date overview of our current understanding of the impact of exercise on fertility, with particular consideration of patient characteristics that may warrant a modification of one’s specific recommendations.

## Materials and methods

An electronic search of Ovid MEDLINE from inception until November 2022 was conducted. Key words included “exercise,” “physical activity,” “infertility,” “fertility,” “menstrual cycle,” “ovulation,” “assisted reproduction,” “in vitro fertilization,” and “polycystic ovary syndrome,” among others. Included studies could follow observational or experimental research designs. Participants could be male or female as long as they were attempting to conceive with or without medical intervention. In reviewing the research identified in the search, 3 main study populations were identified and discussed separately: healthy (presumed fertile) women attempting to conceive, women with diagnosed polycystic ovary syndrome (PCOS), and women undergoing assisted reproduction.

To narrow the focus of this mini-review, it was decided that only studies measuring objective markers of fertility would be included. Such objective markers included pregnancy, ovulation, or levels of ovarian hormones (i.e., estradiol and progesterone) across the menstrual cycle. Thus, studies solely assessing self-reported menstrual regularity or surrogate markers of disease severity (e.g., acne or androgen levels in PCOS) were excluded.

## Defining physical activity and exercise

Physical activity (PA) encompasses all leisure and nonleisure bodily movements produced by skeletal muscle groups that raise energy expenditure over resting; exercise is a subcategory of PA that is structured, repetitive, and purposive toward maintaining or improving fitness levels. PA and exercise intensity can be measured using objective measures, such as heart rate or metabolic equivalents, a measure of energy expenditure (2), or subjective measures, such as self-rated perceived exertion. Table 1 summarizes how exercise can be categorized into mild, moderate, and vigorous intensity using these various measures.

**Table 1:** Comparing exercise intensities using various methods of measurement.

Exercise intensity	METs	HR	Borg rating of perceived exertion (RPE; 6–20 scale)	Subjective experience	Examples
Light	2.0–2.9	30%–39% HR reserve^a^ 57%–63% max HR	9–11	Movement that does not cause adults to sweat or breathe harder; easy to talk	Walking slowly (2.0 METs) Housework while standing (2.0–2.5 METs)
Moderate	3.0–5.9 METs	40%–59% HR reserve 64%–76% max HR	12–13	Causes some sweating and shortness of breath; can only speak short sentences	Brisk walk: 5.0 METs Bicycling (leisurely): 5.5–6.0 METs
Vigorous	≥6.0 METs	60%–89% HR reserve 77%–955% max HR	14–17	Causes considerable sweating and shortness of breath; can only speak 1–2 words	Swimming (leisurely): 6.0 METs Running 9.65 km/h: 9.5–10 METs

HR = heart rate; max = maximum; MET = metabolic equivalent; RPE = rating of perceived exertion.

a Heart rate reserve is calculated by taking the theoretical maximum HR (208 − [0.7 × age]) and subtracting the resting HR (4). MET values refer to the energy expenditure, wherein 1 MET is at rest and 5 METs refer to expending 5 times the amount of energy a person would sitting at rest (2).

## The effect of exercise on fertility

In the general population, moderate-to-vigorous exercise is generally considered beneficial because it reduces the risk of multiple chronic illnesses, such as cardiovascular disease and cancer (3); during pregnancy, regular moderate-to-vigorous exercise is associated with improved maternal and fetal health outcomes (4). From a mental health perspective, regular engagement in exercise has also been shown to have large beneficial effects on both depressive and anxious moods in the general population (5) and, specifically, in women struggling with infertility (6, 7). However, what is less clear-cut is the impact of exercise of varying intensities on fertility among women either attempting and/or struggling to conceive. Therefore, this review considers the existing research examining the impact of exercise on indicators of fertility, specifically rates of ovulation and pregnancy as well as ovarian hormone levels, with specific consideration for 3 separate populations that have been of particular focus in this line of research: presumed healthy women attempting to conceive without medical intervention, women with diagnosed PCOS attempting to conceive without medical intervention, and women with diagnosed infertility pursuing assisted reproduction.

## Healthy women attempting to conceive

A number of studies have examined the effects of PA on fertility in healthy women attempting to conceive (Table 2). For example, an early study by De Souza et al. (8) (2003) compared runners running a mean (±SD) of 32 km/wk with sedentary women, tracking hormonal markers and menstrual cycle characteristics over 3 consecutive cycles. In this study, 58% of regular runners were found to show menstrual cycle abnormalities, including anovulation and an insufficient luteal phase (defined as a luteal phase of <10 days or a peak urinary pregnanediol level of <1.5 Creatine-adjusted μg/mL for ≥3 midluteal days) vs. only 9% of sedentary women. Furthermore, among the ovulatory cycles with a sufficient luteal phase, luteal progesterone levels were lower among runners than among sedentary women. Runners were also found to show lower levels of estrone glucuronide, a urinary metabolite of estradiol, in the late follicular phase, suggesting an inhibitory effect of exercise on follicular maturation. Although the total weekly distance run was not associated with any of the parameters assessed, the net energy balance (i.e., caloric intake minus caloric expenditure), assessed using daily dietary records and activity monitoring, was. Specifically, cycles that were categorized as anovulatory were associated with a lower energy balance than those with other types of cycles. However, cycles characterized by an insufficient luteal phase were found to be associated with an energy balance similar to that of ovulatory menstrual cycles.

**Table 2:** The effects of physical activity in healthy females.

Investigator, year	Participants	Study design	Intervention and PA assessment	Control group	Main findings
Williams et al. (11), 2015	34 premenopausal eumenorrheic women with a normal BMI, all aged between 18 and 30 y	Randomized controlled trial	3 intervention groups, all involving 3 mo of supervised vigorous aerobic exercise 5 d/wk, ranging from 20–75 min, and controlled diet to manipulate caloric intake. Group 1 experienced a mild exercise calorie deficit, group 2 experienced a moderate exercise calorie deficit, and group 3 experienced a severe exercise calorie deficit	The same exercise regimen as intervention groups without an energy deficit	85% of the moderate and severe energy deficit groups experienced at least 1 luteal phase defect in the 3 intervention menstrual cycles. In contrast, only 1 participant in the control group and 1 in the mild deficit group did A trend toward higher rates of anovulation (35%) in moderate and severe deficit groups (*P* = .07) relative to other groups (0)
De Souza et al. (8), 2003	35 eumenorrheic women aged between 18 and 36 y	Cross-sectional study	Comparison of sedentary women (n = 11) and active runners (n = 24) running at least 2 h/wk (average of 32 km/wk	N/A	Of the 3 menstrual cycles assessed, 16% of the running group was deemed anovulatory vs. none of the sedentary women. In addition, 42% of the running group exhibited a luteal phase abnormality (short or insufficient) vs. 9% of the sedentary group. Luteal progesterone was lower in intact ovulatory cycles among runners vs. sedentary women
Wise et al. (9), 2012	3,027 women aged 18–40 y, not receiving any type of fertility treatment	Prospective cohort study	Women reported the average number of hours per week that they engaged in PA during the past year, reporting moderate and vigorous activity separately	N/A	Dose-response relationship between the number of hours of vigorous PA and the time to pregnancy in women with normal BMIs. Time to pregnancy was significantly lower in participants engaging in moderate PA only. Lower fecundability in all women who engaged in vigorous PA
Gudmunds-dottir et al. (10), 2009	3,887 premenopausal women aged 20–45 y	Prospective cohort study	Weekly frequency, intensity, and duration of leisure-time PA were self-reported, and the amount of PA was categorized as either low, moderate, or high on the basis of divisions at the 33rd and 66th percentiles	N/A	Women who exercised every day were 3.2 times more likely to be infertile than inactive women, and exercising to exhaustion was associated with 2.3 times the odds of infertility compared with taking it easy. Exercising for 15–30 or 30–60 min was associated with lower odds of infertility compared to exercising for <15 min

*Note:* BMI = body mass index; N/A = not applicable; PA = physical activity.

The potential for vigorous exercise to negatively impact fertility in healthy women has been confirmed in 2 large prospective studies. The first is by Wise et al. (9) (2012), which found a dose-response relationship between the self-reported number of hours of vigorous exercise and time to pregnancy in women with a normal body mass index (BMI). Specifically, in models adjusting for BMI and a number of health behaviors, women engaging in 2 weekly hours of vigorous exercise were 16% less likely to be pregnant than sedentary women over the same time frame. Among women exercising 3–4 h/wk, this number increased to 27% less likely, whereas ≥5 hours were associated with a 32% reduction in the chances of conception. In contrast, moderate exercise increased the odds of conceiving; for example, 2 hours of moderate activity increased the odds of pregnancy by 15%. The second is a prospective study by Gudmundsdottir et al. (10) (2009) that examined the impact of self-reported exercise in less detail but largely confirmed the findings of Wise et al. (9) (2012). Specifically, the researchers found that women categorized as having a “high” PA index, determined through a combination of PA frequency and intensity, were 1.5 times more likely to experience subfertility or involuntary childlessness compared with women with a “low” PA index.

However, a randomized controlled trial by Williams et al. (11) (2015) provides valuable information about the importance of the energy deficit induced by exercise in determining its deleterious effects on fertility. This study compared 3 intervention groups involving vigorous exercise 5 d/wk and a controlled diet to manipulate calorie intake. The mild energy deficit condition involved a 15% caloric deficit through the modified caloric intake, the moderate deficit condition involved a 30% caloric deficit, and the severe deficit condition involved a 60% caloric deficit. In contrast, the control group increased their caloric intake to ensure that the exercise regimen did not induce a caloric deficit. Hormonal and menstrual cycle length parameters were monitored throughout the 3-month study. Overall, 85% of the women assigned to the moderate and severe caloric deficit groups experienced at least 1 luteal phase defect (defined as a luteal phase of <10 days or a peak urinary pregnanediol level of <5.0 μg/mL). Anovulation also tended to be more prevalent in the moderate and severe energy deficit groups, although this effect did not reach statistical significance, perhaps because of the study’s modest sample size. However, importantly, the rates of both anovulation and luteal phase defects remained low in the mild energy deficit group and the control group. The results suggest that the detrimental effects of vigorous exercise on fertility in healthy-weight women might be countered through increased calorie intake. However, future research directly assessing pregnancy rates in a larger sample is needed to confirm this conclusion.

## Takeaways for healthy women

Although several studies have suggested that frequent engagement in vigorous exercise may have deleterious effects on fertility, the aforementioned research by Williams et al. (11) is encouraging for highly active women wishing to continue their exercise regimen while trying to conceive. On the basis of their findings, women wanting to continue their vigorous exercise regimen while attempting to conceive could be instructed to ensure that on workout days, they increase their caloric intake by approximately the number of calories expended during their exercise session. Practitioners may also consider assessing midluteal progesterone and luteal phase length to rule out the presence of a luteal phase defect. In the event that a defect is detected, the patient could be instructed to either attempt to increase her calorie consumption or replace vigorous exercise with moderate-intensity activity, as defined in Table 1. This modification may be particularly beneficial during the follicular phase to ensure adequate follicular maturation.

## Women with diagnosed PCOS

Polycystic ovary syndrome is a common endocrinopathy involving insulin resistance, menstrual dysfunction, and anovulatory infertility. Lifestyle interventions primarily emphasizing weight loss have recently been found in numerous studies to approximately double live births in women with PCOS (12). However, many of these studies were not eligible to be included in this review because they included an intervention combining diet and exercise, making it impossible to separate the effects of diet and weight loss vs. exercise alone. To our knowledge, only 4 studies have directly examined the impact of exercise alone on ovulation and/or pregnancy outcomes in this population (Table 3) (13). Two key findings stem from this research. First, as seen in the study by Palomba et al. (14) (2008), as little as 30 minutes of vigorous exercise 3 times per week can significantly increase the chances of conception among overweight women with PCOS. One prospective cohort study of 116,671 nurses aged 25–42 years by Rich-Edwards et al. (15) (2002) further emphasizes that engaging in vigorous activity may have greater benefits than moderate activity for anovulation. Specifically, this study found that when considering self-reported PA, each 24-hour increase in vigorous (but not moderate) PA was associated with a 7% reduced risk of ovulatory infertility, an effect that remained after statistically adjusting for BMI. Although their investigation was not specific to PCOS, it is noteworthy that PCOS is the most common cause of ovulatory infertility. The lack of benefit seen in the study by Nybacka et al. (16) (2011), who tailored their exercise intervention to each participant’s baseline fitness level with no specific intensity targets, also supports the specific benefits of vigorous exercise for PCOS.

**Table 3:** Effects of exercise on fertility in women with polycystic ovary syndrome.

Investigator, year	Participants	Study design	Intervention and PA assessment	Control group	Main findings
Clark et al. (13), 1998	87 treatment-seeking obese women with PCOS	A nonrandomized controlled trial	1-h low-impact group exercise session weekly plus 2 additional sessions alone encouraged, for 6 mo Weekly information sessions about healthy eating	Participants who dropped out of the study prematurely (n = 20/87)	Increased ovulation (90% vs. 0) and live birth rate (67% vs. 0) in completers vs. drop-outs
Nybacka et al. (16), 2011	43 overweight or obese women with PCOS, aged 18–40 y	Randomized parallel design	A 4-mo exercise program with a physical therapist—content, and frequency tailored to patient preference, with or without dietary management	4 mo of dietary management (600 kcal/d caloric reduction)	69% of participants demonstrated improved menstruation, and 35% demonstrated ovulation. All 3 groups (diet, exercise, and both) exhibited similar improvements, despite the diet-only group showing the greatest reduction in free testosterone
Palomba et al. (14), 2008	40 obese, infertile, and anovulatory patients with PCOS were referred for treatment	Nonrandomized controlled trial (participants chose the desired condition)	24 wk of aerobic exercise on a stationary cycle for 30 min, 3 d/wk. Target intensity 60%–70% VO_2_ max	A hypocaloric and hyperproteic diet for 24 wk	Higher rate of ovulation (65% vs. 25%) and spontaneous pregnancy (35% vs. 10%) in the exercise group compared with the diet group
Thomson et al. (17), 2008	154 sedentary and overweight or obese women with PCOS, aged 18–41 y	Randomized parallel design	20 wk of either DO, DA (5 weekly sessions of 45-min jogging at 60%–80% HR_max_), or DC (3 weekly jogging sessions plus 2 resistance training sessions)	The comparison group was diet-only (hyperproteic and hypocaloric) or diet and aerobic plus resistance	Cardiometabolic parameters improved similarly across all 3 groups, as did FAI. DA + DC lost more weight than DO. DA had a greater number of ovulatory cycles than DO (3.1 vs. 1.3), with DC being in between, with 2.7 ovulatory cycles

*Note:* DA = dieting and aerobic exercise; DC = diet and combined aerobic and resistance exercise; DO = dieting; HR_max_ = maximum heart rate; FAI = free androgen index; PA = physical activity; PCOS = polycystic ovary syndrome; VO_2_ max = maximal oxygen consumption.

Importantly, the benefits of exercise for fertility in women with PCOS do not appear to be fully explained by reductions in weight. This is supported by the fact that in the trial by Palomba et al. (14) (2008), the exercise condition outperformed the diet condition in terms of ovulation rates despite achieving less weight loss. This conclusion is also supported by the fact that in the aforementioned large prospective study by Rich-Edwards et al. (15), a strong association between vigorous exercise and a lowered risk of ovulatory infertility remained after including BMI as a covariate.

Several studies have examined the impact of resistance training on metabolic parameters in women with PCOS (discussed below); however, only 1 of the studies in Table 3 addressed the benefits of resistance training for fertility. Unfortunately, its results are not straightforward. Specifically, the study by Thomson et al. (17) (2008) compared the effects of diet plus vigorous aerobic exercise vs. diet plus vigorous aerobic exercise plus resistance training and found that the diet plus vigorous aerobic exercise combination resulted in slightly more ovulatory menstrual cycles than those with the regimen combining both types of exercise. Hence, this study suggests that adding resistance training to an exercise regimen that already includes vigorous aerobic exercise may not be of benefit. However, whether resistance training alone is as beneficial as vigorous aerobic exercise alone for individuals with PCOS remains unknown.

Although the above-described studies are, to our knowledge, the only trials to specifically examine the effect of exercise (alone) on pregnancy, ovulation, or ovarian hormones in women with PCOS, we thought it relevant to mention a recent systematic review and meta-analysis examining the health benefits of exercise for PCOS more broadly (18). This review identified 33 exercise intervention trials in this population. The median exercise frequency of the interventions was 3 times per week of moderate and/or vigorous intensity; across all studies, the average baseline BMI fell within the overweight or obese category. Overall results revealed that both vigorous aerobic exercise and resistance training led to the greatest improvements in insulin resistance and that vigorous aerobic exercise had the greatest benefits for body mass. Of the 16 studies measuring outcomes relevant to reproduction (e.g., testosterone, sex hormone binding globulin, menstrual cycle length, and pregnancy), 8 observed favorable changes in 1 or more reproductive parameters with exercise. Resistance training appeared to have the greatest reproductive benefits relative to aerobic activity alone, particularly in relation to the free androgen index. For example, 1 small randomized controlled trial directly compared the effects of 3 weekly sessions of high-intensity interval training (4 × 4 minutes of high-intensity aerobic exercise) with strength training (3 sets of 10 repetitions × 8 resistance exercises) and found that strength training led to reductions in antimüllerian hormone and the free androgen index and increases in sex hormone binding globulin, none of which were seen in the high-intensity interval training group (19). The meta-analysis further revealed that adherence to a long-term regimen was an important contributor to outcomes, with ≥50 hours being associated with the greatest reduction in the free androgen index. This suggests that long-term resistance training appears to be best suited for improving hyperandrogenicity in PCOS.

## Takeaways for women with PCOS

Although the number of studies directly testing the effect of exercise on ovulation or pregnancy rates in women with PCOS is small, there are nonetheless a few key conclusions that can be made on the basis of the existing research. First, moderate aerobic exercise appears to be insufficient to induce any beneficial effects on fertility in this population. Instead, vigorous aerobic exercise and perhaps resistance training appear to be the most directly related to reproductive benefits. Second, the maximum benefits of an exercise regimen for hyperandrogenicity may take up to 50 training hours (i.e., roughly 1 month of moderate-to-vigorous intensity exercise) to appear (17). Therefore, patients should be encouraged to develop an exercise program that they can adhere to over the long term. Third, exercise benefits fertility through mechanisms other than weight loss. Thus, women should be assured that, even in the absence of any measurable changes in weight, their engagement in exercise is likely to improve their chances of conceiving. This last finding suggests that healthy-weight women with PCOS may also benefit from vigorous exercise, although there is a lack of studies directly testing this.

## Exercise and assisted reproduction outcomes

The available research in the field of exercise and assisted reproduction outcomes appears to suggest that engagement in regular PA before and during an in vitro fertilization (IVF) cycle either has no effect on cycle success or may have a small beneficial effect (Table 4). Specifically, in a longitudinal study by Sõritsa et al. (20) (2020) that used both objective accelerometry and subjective self-reported PA, neither PA duration nor intensity was associated with fertility-related outcomes. Similarly, in a moderately large prospective study by Gaskins et al. (21) (2016) (n = 275), there was no overall effect of PA on IVF outcomes; however, patient BMI was found to moderate this relationship such that increased participation in moderate-to-vigorous PA was associated with an increased likelihood of live birth among healthy-weight women but not in overweight women. Total recreational and moderate-intensity PA were associated positively with the number of retrieved oocytes and pregnancy outcomes, respectively, in a smaller cohort study by Prémusz et al. (6) (2021). Studies by Kucuk et al. (22) (2010) and Palomba et al. (23) (2014) generated similar findings in which patients who reported higher levels of PA were more likely to have an increased live birth rate.

**Table 4:** Effects of physical activity on assisted reproductive technology outcomes.

Investigator, year	Participants	Study design	PA assessment	Main findings
Gaskins et al. (21), 2016	275 women between the ages of 18 and 46 y undergoing IVF	Prospective cohort study	PA was measured using a questionnaire, women reported average time per week spent in PA	In healthy-weight women, moderate-to-vigorous activity was positively associated with live births (i.e., 27% success rate in the lowest quintile vs. 47% in the highest). This association was not significant in overweight women
Kucuk et al. (22), 2010	131 women undergoing assisted reproduction	Prospective cohort study	PA frequency and duration in a normal week were assessed via a questionnaire	Women categorized in the moderate activity group achieved a higher clinical pregnancy rate (54% vs. 34%) and live birth rate (48% vs. 22%) than those in the low-activity group
Morris et al. (24), 2006	2,232 couples from 3 IVF clinics	Prospective cohort study	A questionnaire assessing type (walking, cardiovascular, and other), duration per week, and the number of years of participation in this activity	No linear association between total baseline PA and outcomes Some significant findings for categorical analyses: women who engaged in PA for 4+ h/wk, for 1–9 y before IVF were 40% less likely to become pregnant than non-exercisers. However, this was not the case for women engaging in 4+ h of PA between 10 and 20 y before IVF
Sõritsa et al. (20), 2020	101 infertile women undergoing IVF treatment	Longitudinal study	PA was measured for 14 d each using accelerometry before IVF, during IVF at implantation time, and immediately after embryo transfer	No effect of PA before or during IVF on clinical pregnancies or live births
Palomba et al. (23), 2014	216 obese women with PCOS undergoing IVF or ICSI	Prospective cohort study	Self-reported type and frequency of PA using the global PA questionnaire	Women engaging in regular PA had a significantly higher pregnancy rate (39% vs. 16%) and total birth rate (24% vs. 7%) compared with women who did not exercise
Prémusz et al. (6), 2021	28 women between the ages of 18 and 40 y, BMI between 18 and 38, undergoing IVF	A cross-sectional observational cohort study	Self-reports on the type and frequency of PA using the global PA questionnaire, validated using objective measures	Total recreational PA was positively correlated with oocytes retrieved (r = 0.34). Time spent on moderate PA per week positively predicted live births. Vigorous PA was unrelated to pregnancy outcomes

*Note:* BMI = body mass index; ICSI = intracytoplasmic sperm injection; IVF = in vitro fertilization; PA = physical activity; PCOS = polycystic ovary syndrome.

The only study to observe a negative effect of PA on IVF outcomes was a large prospective study of >2,000 couples undergoing IVF by Morris et al. (24) (2006); however, the results were somewhat conflicting. On the one hand, there was no linear association between PA and IVF success. The researchers then investigated different categories of exercisers, separated by both total exercise duration per week as well as the number of years during which the participant had been engaging in that activity. In those analyses, women who had been engaging in 4+ hours of PA per week for 1–9 years were significantly less likely to achieve pregnancy than women who reported no exercise. However, surprisingly, this was not the case for women who had been engaging in the same regimen for a longer time (10–20 years). This finding is somewhat difficult to explain and raises the question of whether these categories of women differed in other important yet unmeasured ways, such as the couple’s infertility-related diagnosis.

## Takeaways for women undergoing assisted reproduction

For the most part, the existing literature suggests that PA before and during IVF has either no effect or a positive effect on pregnancy outcomes. Combined with results from the research described in the previous sections, these findings suggest that although vigorous exercise can negatively impact fertility by interfering with ovulation in the context of a natural menstrual cycle, the negative effects of vigorous exercise are no longer apparent when ovulation is carefully controlled through pharmacologic manipulation. Thus, these results suggest that active women can be advised to continue their normal activities during assisted reproduction treatment cycles.

## Conclusions

Our review of the literature suggests that recommendations regarding PA while trying to conceive should be tailored on the basis of specific patient characteristics. Specifically, overweight and obese women with PCOS should be encouraged to engage in vigorous aerobic exercise or resistance training to experience their insulin-sensitizing effects; similar recommendations can likely be applied to lean women with PCOS, although future research is needed to confirm this. In the case of women undergoing assisted reproduction for other diagnoses, exercise likely has little to no impact on treatment outcomes—individuals can therefore continue their regular exercise regimen throughout treatment.

However, in the case of healthy women who are trying to conceive, the potential for regular vigorous exercise to negatively impact fertility should be considered. That is, the exercise regimen of women presenting for evaluation of subfertility should be assessed, and the presence of anovulation and/or a luteal phase defect caused by vigorous exercise should be considered. When anovulation and/or a luteal phase defect are detected, the patient could be encouraged to increase her caloric intake to avoid an exercise-induced deficit or, when necessary, to replace vigorous exercise with moderate-intensity exercise for a time to see whether menstrual cycles normalize. However, in light of the considerable mental and physical health benefits of regular exercise, we would advocate against indiscriminately discouraging highly active women from engaging in vigorous exercise while trying to conceive.

These recommendations should be considered in light of several limitations characterizing the available research on exercise and fertility. For example, many studies are observational in nature and vary in their consideration of potential confounding variables. Several of the included studies also had a small sample size, raising concerns about statistical power and generalizability. The definition of “moderate” or “vigorous” exercise sometimes varied between studies and was sometimes assessed via self-report. In addition to these methodological limitations, several clinical questions remain unanswered. For example, further research is needed to inform clinical recommendations for women experiencing nonovulatory infertility and not undergoing assisted reproduction. Although levels of PA have not been associated with prevalence rates of endometriosis (25), no research to date has examined PA and endometriosis severity or pregnancy rates in women with the condition. In addition, studies directly testing the effect of resistance training on ovulation and pregnancy are needed, particularly in women with PCOS.

## References

[1] Practice Committee of the American Society for Reproductive Medicine (2020). Definitions of infertility and recurrent pregnancy loss: a committee opinion. Fertil Steril.

[2] American College of Sports Medicine (2017).

[3] Warburton D.E.R., Bredin S.S.D. (2017). Health benefits of physical activity: a systematic review of current systematic reviews. Curr Opin Cardiol.

[4] Mottola M.F., Davenport M.H., Ruchat S.M., Davies G.A., Poitras V.J., Gray C.E. (2018). 2019 Canadian guideline for physical activity throughout pregnancy. Br J Sports Med.

[5] Saxena S., Van Ommeren M., Tang K.C., Armstrong T.P. (2005). Mental health benefits of physical activity. J Ment Health.

[6] Prémusz V., Makai A., Perjés B., Máté O., Hock M., Ács P. (2021). Multicausal analysis on psychosocial and lifestyle factors among patients undergoing assisted reproductive therapy–with special regard to self-reported and objective measures of pre-treatment habitual physical activity. BMC Public Health.

[7] Rebar A.L., Stanton R., Geard D., Short C., Duncan M.J., Vandelanotte C. (2015). A meta-meta-analysis of the effect of physical activity on depression and anxiety in non-clinical adult populations. Health Psychol Rev.

[8] De Souza M.J., Van Heest J., Demers L.M., Lasley B.L. (2003). Luteal phase deficiency in recreational runners: evidence for a hypometabolic state. J Clin Endocrinol Metab.

[9] Wise L.A., Rothman K.J., Mikkelsen E.M., Sørensen H.T., Riis A.H., Hatch E.E. (2012). A prospective cohort study of physical activity and time to pregnancy. Fertil Steril.

[10] Gudmundsdottir S.L., Flanders W.D., Augestad L.B. (2009). Physical activity and fertility in women: the North-Trøndelag Health Study. Hum Reprod.

[11] Williams N.I., Leidy H.J., Hill B.R., Lieberman J.L., Legro R.S., De Souza M.J. (2015). Magnitude of daily energy deficit predicts frequency but not severity of menstrual disturbances associated with exercise and caloric restriction. Am J Physiol Endocrinol Metab.

[12] Hunter E., Avenell A., Maheshwari A., Stadler G., Best D. (2021). The effectiveness of weight-loss lifestyle interventions for improving fertility in women and men with overweight or obesity and infertility: a systematic review update of evidence from randomized controlled trials. Obes Rev.

[13] Clark A.M., Thornley B., Tomlinson L., Galletley C., Norman R.J. (1998). Weight loss in obese infertile women results in improvement in reproductive outcome for all forms of fertility treatment. Hum Reprod.

[14] Palomba S., Giallauria F., Falbo A., Russo T., Oppedisano R., Tolino A. (2008). Structured exercise training programme versus hypocaloric hyperproteic diet in obese polycystic ovary syndrome patients with anovulatory infertility: a 24-week pilot study. Hum Reprod.

[15] Rich-Edwards J.W., Spiegelman D., Garland M., Hertzmark E., Hunter D.J., Colditz G.A. (2002). Physical activity, body mass index, and ovulatory disorder infertility. Epidemiology.

[16] Nybacka Å., Carlström K., Ståhle A., Nyrén S., Hellström P.M., Hirschberg A.L. (2011). Randomized comparison of the influence of dietary management and/or physical exercise on ovarian function and metabolic parameters in overweight women with polycystic ovary syndrome. Fertil Steril.

[17] Thomson R.L., Buckley J.D., Noakes M., Clifton P.M., Norman R.J., Brinkworth G.D. (2008). The effect of a hypocaloric diet with and without exercise training on body composition, cardiometabolic risk profile, and reproductive function in overweight and obese women with polycystic ovary syndrome. J Clin Endocrinol Metab.

[18] Patten R.K., Boyle R.A., Moholdt T., Kiel I., Hopkins W.G., Harrison C.L. (2020). Exercise interventions in polycystic ovary syndrome: a systematic review and meta-analysis. Front Physiol.

[19] Almenning I., Rieber-Mohn A., Lundgren K.M., Shetelig Løvvik T., Garnæs K.K., Moholdt T. (2015). Effects of high intensity interval training and strength training on metabolic, cardiovascular and hormonal outcomes in women with polycystic ovary syndrome: a pilot study. PLoS One.

[20] Sõritsa D., Mäestu E., Nuut M., Mäestu J., Migueles J.H., Läänelaid S. (2020). Maternal physical activity and sedentary behaviour before and during in vitro fertilization treatment: a longitudinal study exploring the associations with controlled ovarian stimulation and pregnancy outcomes. J Assist Reprod Genet.

[21] Gaskins A.J., Williams P.L., Keller M.G., Souter I., Hauser R., Chavarro J.E. (2016). Maternal physical and sedentary activities in relation to reproductive outcomes following IVF. Reprod Biomed Online.

[22] Kucuk M., Doymaz F., Urman B. (2010). Effect of energy expenditure and physical activity on the outcomes of assisted reproduction treatment. Reprod Biomed Online.

[23] Palomba S., Falbo A., Chiossi G., Orio F., Tolino A., Colao A. (2014). Low-grade chronic inflammation in pregnant women with polycystic ovary syndrome: a prospective controlled clinical study. J Clin Endocrinol Metab.

[24] Morris S.N., Missmer S.A., Cramer D.W., Powers R.D., McShane P.M., Hornstein M.D. (2006). Effects of lifetime exercise on the outcome of in vitro fertilization. Obstet Gynecol.

[25] Ricci E., Viganò P., Cipriani S., Chiaffarino F., Bianchi S., Rebonato G. (2016). Physical activity and endometriosis risk in women with infertility or pain: systematic review and meta-analysis. Medicine.

